# Whole-body water mass and kidney function: a Mendelian randomization study

**DOI:** 10.3389/fendo.2024.1336142

**Published:** 2024-04-03

**Authors:** Xuejiao Wei, Mengtuan Long, Zhongyu Fan, Yue Hou, Liming Yang, Zhihui Qu, Yujun Du

**Affiliations:** Department of Nephrology, The First Hospital of Jilin University, Changchun, China

**Keywords:** chronic kidney disease, kidney function, body water content, Mendelian randomization, whole-body water mass

## Abstract

**Background:**

The morbidity and mortality of chronic kidney disease (CKD) are increasing worldwide, making it a serious public health problem. Although a potential correlation between body water content and CKD progression has been suggested, the presence of a causal association remains uncertain. This study aimed to determine the causal effect of body water content on kidney function.

**Methods:**

Genome-wide association study summary data sourced from UK Biobank were used to evaluate single-nucleotide polymorphisms (SNPs) associated with whole-body water mass (BWM). The summary statistics pertaining to kidney function were extracted from the CKDGen consortium. The primary kidney function outcome measures included estimated glomerular filtration rate (eGFR), albuminuria, CKD stages 3–5, and rapid progression to CKD (CKDi25). Two-sample Mendelian randomization (MR) analysis estimated a potential causal relationship between the BWM and kidney function. The inverse variance weighted MR method was used as the primary analysis, accompanied by several sensitive MR analyses.

**Results:**

The increase of BWM exhibited a correlation with a reduction in eGFR (β = −0.02; *P* = 6.95 × 10^−16^). Excluding 13 SNPs responsible for pleiotropy (*P* = 0.05), the increase of BWM was also associated with the decrease of the ratio of urinary albumin to creatinine (β = −0.16; *P* = 5.91 × 10^−36^). For each standard deviation increase in BWM, the risk of CKD stages 3–5 increases by 32% (OR, 1.32; 95% CI, 1.19–1.47; *P* = 1.43 × 10^−7^), and the risk of CKDi25 increases by 22% (OR, 1.22; 95% CI, 1.07–1.38; *P* = 0.002).

**Conclusion:**

The increase of BWM is associated with impaired kidney function. Proactively managing body water content is of great significance in preventing the progression of CKD.

## 1 Introduction

Chronic kidney disease (CKD) is a long-term, progressive renal disorder characterized by a gradual decline in kidney function, leading to the ineffective filtration of waste products and excess fluids (1). Edematous status is a common clinical problem among CKD patients. Expanded volume, even in the absence of obvious edema, can cause hypertension, pulmonary congestion, and heart failure (2, 3). Managing body water content is a part of CKD treatment, which includes limiting salt and fluid intake, and getting diuretics and dialysis to remove excess waste products and fluid. Meanwhile, monitoring body water content can be used to track the progression of CKD. Excessive water retention may be linked to lower glomerular filtration rate, tubular dysfunction, and renal fibrosis, all of which can affect the progression of CKD (4). However, the causal relationship between body water content and CKD progression is still unclear.

The clinical evaluation of edematous status is relatively difficult, and bioelectrical impedance analysis (BIA) techniques are often introduced to assessment the liquid status. The ratio of extracellular water (ECW) to total body water (TBW) may indicate volume overload (5). However, the ECW/TBW might not be the most optimal indicator of volume overload due to the change of the composition of intracellular water (6). Compared with clinical evaluation, BIA greatly improved the identification of fluid overload in CKD patients (7, 8). The application of BIA has been proposed to detect subclinical edema and changes in body composition during hemodialysis in CKD patients (9, 10). The research conducted by Ohashi et al. indicated that volume overload occurring in malnourished and elderly patients with CKD is associated with adverse renal outcomes and all-cause mortality (11). Previous studies have demonstrated that the progressive expansion of water in CKD patients is independently related to the poor renal outcomes (12). Han et al. found that fluid overload is the decisive factor of cardiac structure and functional impairment in patients with type 2 diabetes mellitus and advanced CKD without dialysis (13, 14).

Mendelian randomization (MR) analysis employs genetic variations strongly correlated with exposure factors as instrumental variables to deduce causal relationships with the outcome (15). It has the benefit of avoiding confounding biases in observational studies (16). Two-sample MR analysis uses genome-wide association study (GWAS) summary data to estimate the causal effect of exposure on outcomes without having to analyze individual data. Given the current lack of evidence on the causal relationship between body water content and the development of CKD, this study used two-sample MR analysis to explore the impact of edematous status on kidney function in patients with CKD.

## 2 Methods

### 2.1 Data source of whole-body water mass

We utilized whole-body water mass (BWM) as an instrumental variable to identify genetic variations related to body water content. The BWM-related GWAS data were obtained from the UK Biobank, including a comprehensive cohort of 454,888 participants of European ancestry. The participants used the BIA device for body water measurement. BWM data were obtained using the Tanita BC418MA body composition analyzer, with measurements of whole-body bioimpedance, accurate to 0.1 kg. The population in the UK Biobank cohort were recruited through 22 assessment centers in the United Kingdom, primarily collecting the disease and lifestyle information and genotype data. All volunteers signed an informed consent, and the study was approved by the north west multicenter research ethics committee. The details of the population characteristics and protocol are available from the UK Biobank (http://www.ukbiobank.ac.uk/about-biobank-uk/).

### 2.2 Data source of kidney function

The GWAS data related to estimated glomerular filtration rate (eGFR), albuminuria, and CKD from the meta-analysis were obtained from the Chronic Kidney Disease Genetics (CKDGen) Consortium as measures of kidney function. The GFR was estimated using the Chronic Kidney Disease Epidemiology Collaboration formula (for individuals >18 years of age) (17), or the Schwartz formula (for individuals ≤18 years of age) (18). The GFR was estimated in the GWAS (n = 765,348) using serum creatinine, the main biomarker to quantify kidney function and define CKD, including mostly Europeans (n = 567,460) and a few Asians and multiple ancestries (19). Albuminuria was measured by the albumin-to-creatinine ratio (UACR), measured in overnight or 24-h urine collections. The GWAS data for UACR were analyzed specifically within participants of European ancestry (20). CKD stages 3–5 were defined as eGFR <60 mL/min/1.73 m^2^. The GWAS data for CKD stages 3–5 include 23 cohorts of European ancestry (n = 480,698; 41,395 cases and 439,303 controls) (19). Furthermore, to better reflect the dynamic effects of body water content on CKD and kidney function, we selected one additional endpoint: rapid progression to CKD (CKDi25), defined as a decline in eGFR by ≥25% from baseline while progression from non-CKD to CKD. The GWAS data for CKDi25 include 42 cohorts of European ancestry (21). **Table 1** displays the summary of sources for kidney function data.

**Table 1:** The summary of sources for kidney function data.

Trait	Ethnicity	Participants included in analysis	Outcome data sources	References
eGFR	Mixed	567,460 Europeans, 165,726 East Asians, 13,842 African-Americans, 13,359 South Asians and 4,961 Hispanics	CKDGen consortium	(19)
UACR	European	5,825 cases and 46,061 controls	CKDGen consortium	(20)
CKD stages 3–5	European	41,395 cases and 439,303 controls	CKDGen consortium	(19)
CKDi25	European	19,901 cases and 175,244 controls	CKDGen consortium	(21)

CKDGen consortium publishes summary-level data in the form of meta-analyses, with age and gender as covariates (CKDGEN Meta-Analysis datasets (uni-freiburg.de)). All participants provided informed consent, and the local ethics committee approved the study (22).

### 2.3 Selection of instrumental variables

First, single-nucleotide polymorphism (SNP) strongly associated with exposure factors which were selected as the instrumental variables, and the filtering condition was *P* < 5 × 10^−8^. Secondly, the selected genetic instruments should meet the independence and linkage disequilibrium (LD) effect, with an LD parameter (r^2^) of <0.001 and of <1 MB from the genetic distance. Additionally, in order to ensure the strength of correlation between instrumental variables and exposure factors, we screened instrumental variables with F statistic >10. The F value was estimated using the formula F = R^2^(N−2)/(1−R^2^), where N represents the sample size in GWAS data and R^2^ represents the degree to which instrumental variables explain the exposure factors (23). The specific formula of R^2^ is as follows: R^2 = ^2 × (1−EAF) × EAF × β^2^, where EAF is the effect allele frequency and β is the effect size of SNP on exposure factors (23). Thirdly, we examined whether exposure-related SNPs were associated with potential risk factors for kidney function using PhenoScanner, an extended tool that associates human genotypes with phenotypes (www.phenoscanner.medschl.cam.ac.uk) (24).

**Figure 1:**
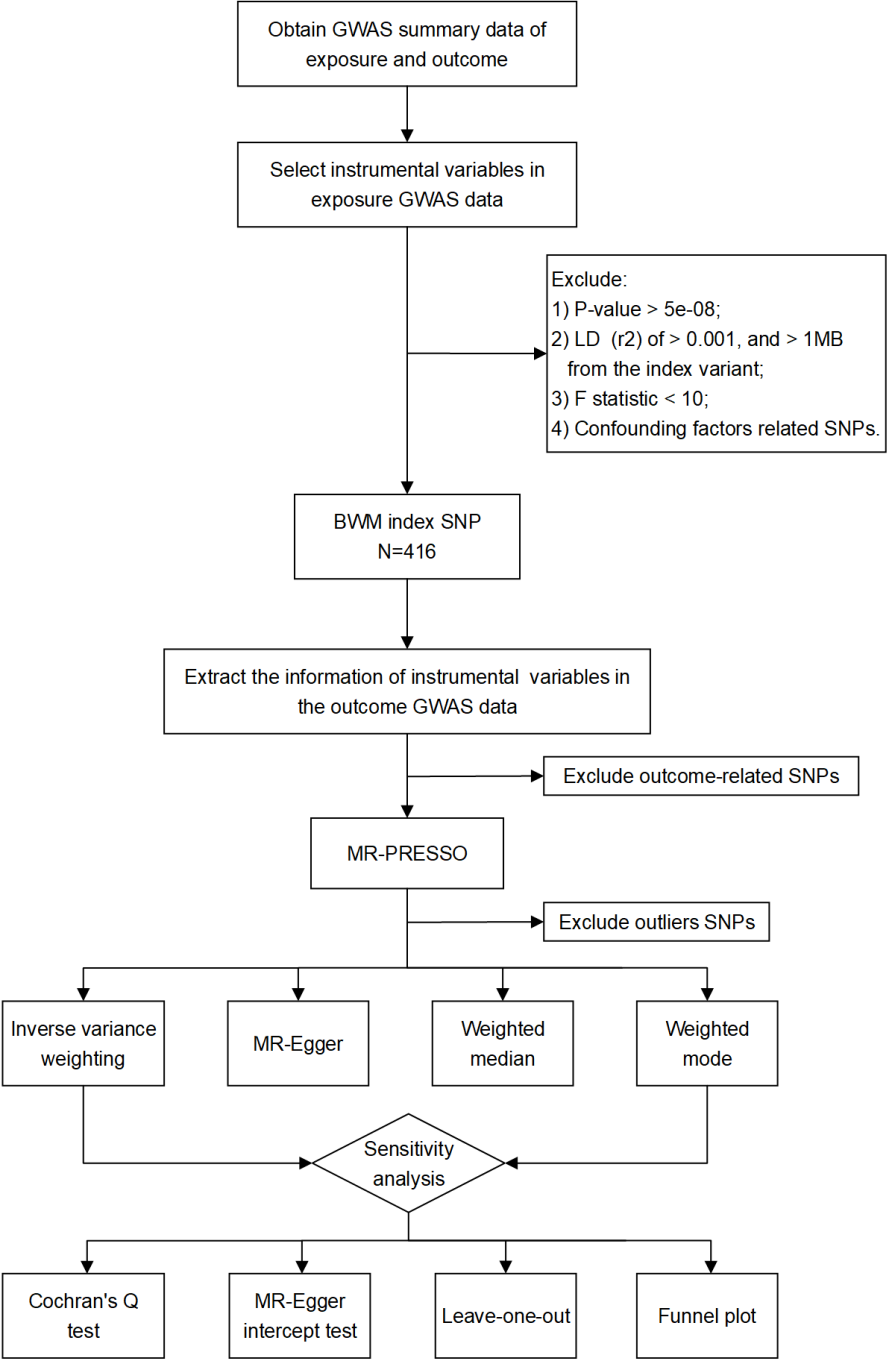
Study flow chart of the MR analyses revealing the causal relationship between BWM and kidney function.

In the UK Biobank dataset, 565 SNPs were associated with BWM at a threshold of *P* < 5 × 10^−8^ and r^2^ < 0.001. Following checking of the potential confounding factors, such as obesity, body mass index, hyperlipidemia, hypertension, diabetes, drinking, and smoking, 416 SNPs remained for subsequent analysis (**Supplementary Table S1**). Next, these SNPs were aligned for effect alleles, unreconciled palindromic SNPs were removed, and the remaining SNPs were subjected to MR analyses. These SNPs’ F statistics surpassed the normal threshold of 10, indicating strong instrument variables. The data files used are provided as **Supplementary Tables S2–S5**. The study flowchart is presented in **Figure 1**.

### 2.4 Mendelian randomization analyses

Two-sample inverse variance weighted (IVW) is used as the primary method for estimating causality. The IVW method assumes that all genetic variations are effective instrumental variables and have a strong ability to detect causality. Particularly, it requires that genetic variations only affect the outcome through the exposure in the study (the intercept is limited to zero) (25). Moreover, we also use MR–Egger, weighted median, and weighted mode to improve the estimated value of IVW and test the reliability and stability of the MR results. In contrast to IVW, MR–Egger regression only needs to satisfy the assumption that instrumental variable pleiotropy remains independent of the association between the instrumental variable and the exposure (26). The weighted median method can still provide effective causal inference even when more than 50% of genetic instruments are invalid (27). Weighted mode analysis relaxes the pleiotropic hypothesis and can still calculate the estimated value when the genetic variation is more than 50% (28). MR analyses were performed for each SNP provided by the GWAS data in turn. The stability and reliability of the causal relation between BWM and kidney function were established when the four different MR methods described above produced similar estimates of the causal effect.

To ensure the accuracy and reliability of the MR results, we subsequently evaluated the directional pleiotropy using the MR–Egger intercept test and leave-one-out analysis. The MR Pleiotropy RESidual Sum and Outlier (MR-PRESSO) method detects and corrects for pleiotropy by removing “outliers”, thereby narrowing the confidence intervals. Cochran’s Q test for identifying heterogeneity. All analyses were performed in R version 4.2.3 using the TwoSampleMR and MRPRESSO packages.

## 3 Result

### 3.1 Total causal effects of BWM on kidney function

**Figure 2:**
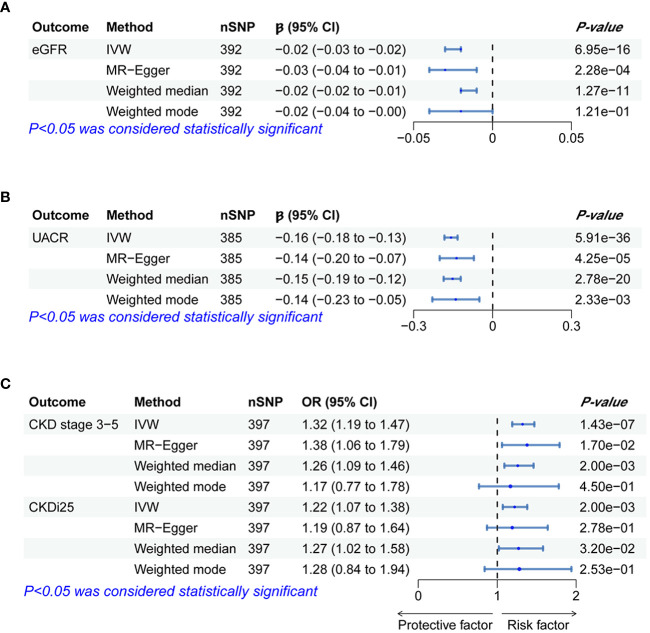
Forest plots of significant and nominal significant estimates from genetically predicted BWM on **(A)** eGFR; **(B)** UACR; **(C)** CKD stage 3-5 and CKDi25.

**Figure 3:**
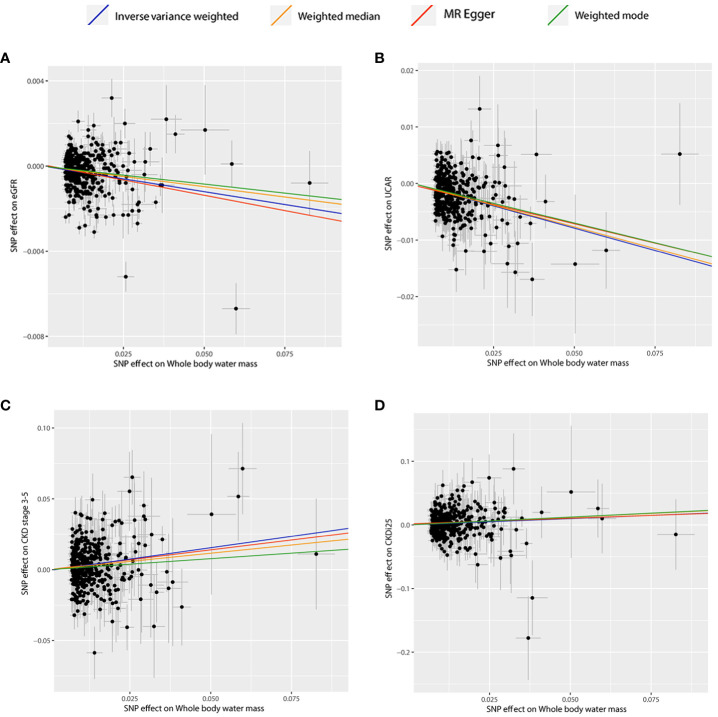
Scatter plots of significant and nominal significant estimates from genetically predicted BWM on **(A)** eGFR; **(B)** UACR; **(C)** CKD stage 3-5; **(D)** CKDi25.

**Table 2** presents the estimated causal effects of BWM on eGFR, UACR, CKD stages 3–5, and CKDi25 in MR analyses. **Figure 2** shows the forest plots of the estimated values for each outcome using different MR methods. **Figure 3** provides scatter plots of SNP–outcome association and SNP–BWM association and visualizes the causal effect estimation of each SNP on kidney function. Leave-one-out plots and funnel plots are provided in **Supplementary Figures S1** and **S2**.

**Table 2:** MR analyses of causal associations of BWM with kidney function.

Trait	nSNP	Ethnicity	IVW		MR–Egger		Weighted median		Weighted mode	
			β or OR*P* (95% CI)		β or OR*P* (95% CI)		β or OR*P* (95% CI)		β or OR*P* (95% CI)	
eGFR	392	Mixed	β = −0.02 (−0.03 to −0.02)	6.95 × 10^−16^	β = −0.03 (−0.04 to −0.01)	2.88 × 10^-4^	β = −0.02 (−0.02 to −0.01)	1.27 × 10^−11^	β = −0.02 (−0.04 to −0.00)	0.12
UACR	385	European	β = −0.16 (−0.18 to −0.13)	5.91 × 10^−36^	β = −0.14 (−0.20 to −0.07)	4.25 × 10^-5^	β = −0.15 (−0.19 to −0.12)	2.78 × 10^−20^	β = −0.14 (−0.23 to −0.05)	2.33×10^−3^
CKD Stages 3–5	397	European	OR = 1.32 (1.19 to 1.47)	1.43 × 10^−7^	OR = 1.38 (1.06 to 1.79)	0.02	OR = 1.26 (1.09 to 1.46)	2.10 × 10^−3^	OR = 1.17 (0.77 to 1.77)	0.45
CKDi25	397	European	OR = 1.22 (1.07 to 1.38)	2.00 × 10^−3^	OR = 1.19 (0.87 to 1.64)	0.28	OR = 1.27 (1.02 to 1.58)	0.03	OR = 1.28 (0.84 to 1.94)	0.25

### 3.2 BWM and eGFR

The IVW analysis examining the association between BWM and eGFR provides strong evidence (β = −0.02, *P* = 6.95 × 10^−16^). The MR–Egger intercept test has not observed directional pleiotropy (*P* = 0.52). However, significant heterogeneity was observed (*P* of Cochran’s Q < 0.05), prompting the preference for the random effect model of IVW to enhance the robustness of the results. In the leave-one-out analysis, β ranged from −0.025 to −0.023, indicating that the overall estimate was unaffected by a single SNP. Furthermore, both MR–Egger (β = −0.03; *P* = 2.28 × 10^−4^) and weight median (β = −0.02; *P* = 1.27 × 10^−11^) consistently support the increase of BWM which is related to the reduction of eGFR. However, due to the limited efficacy, the weighted mode (β = −0.02; *P* = 0.12) method has no statistical significance.

### 3.3 BWM and UACR

MR analyses provide favorable evidence supporting a robust correlation between the increase in BWM and a decrease in UACR. Heterogeneity analysis revealed a significance level of *P* < 0.05, leading to the preference for the random effect model of IVW for enhanced reliability. The MR–Egger intercept test detected directional pleiotropy (*P* = 0.045). MR-PRESSO was used to identify and remove 13 outlier SNPs primarily responsible for pleiotropy (rs113978196, rs114949263, rs1374370, rs143384, rs2101975, rs281385, rs2885697, rs34055910, rs34879158, rs4899012, rs6693481, rs67551338, rs77165542), subsequently calculating the causal estimate. The correlation of IVW analysis is highly significant (β = −0.16, *P* = 5.91 × 10^−36^). MR–Egger (β = −0.14; *P* = 4.25 × 10^−5^), weighted median (β = −0.15; *P* = 2.78 × 10^−20^), and weighted mode (β = −0.14; *P* = 2.33 × 10^−3^) analyses also support the assertion that an increase in BWM is associated with a decrease in UACR. In the leave-one-out analysis following removing outliers, β ranged from −0.161 to −0.155, indicating that the overall estimated value is not affected by a single SNP.

### 3.4 BWM and CKD stages 3–5

In the IVW MR analysis, each standard deviation increase in BWM was associated with a 32% elevation in the risk of CKD stages 3–5 (OR, 1.32; 95%CI, 1.19–1.47; *P* = 1.43 × 10^-7^). The directional pleiotropy was not detected by the MR–Egger intercept test (*P* = 0.73). Heterogeneity analysis found that *P* < 0.05 is preferred for the random effect model of IVW. In the leave-one-out analysis, the estimated values ranged from 1.30 (95% CI, 1.17–1.43) to 1.34 (95% CI, 1.20–1.48). Consistent with IVW analysis, both MR–Egger (OR, 1.38; 95% CI, 1.06–1.79) and weighted median (OR, 1.26; 95% CI, 1.09–1.46) analyses supported that BWM is a potential risk factor for CKD stages 3–5. However, weighted mode (OR, 1.17; 95% CI, 0.77–1.77) analysis is not statistically significant.

### 3.5 BWM and CKDi25

The estimated causal effect of BWM on CKDi25 closely aligns with that observed for CKD stages 3–5 in both direction and magnitude. For every standard deviation increase in BWM, the risk of CKDi25 increases by 22% (OR, 1.22; 95% CI, 1.07–1.38; *P* = 2.00 × 10^−3^). No directional pleiotropy (*P* = 0.89) and heterogeneity were detected (*P* = 0.59). In the leave-one-out analysis, the OR ranges from 1.20 (95% CI, 1.06–1.36) to 1.23 (95% CI, 1.09–1.40). In line with the IVW method, the weighted median (OR, 1.27; 95% CI, 1.02–1.58; *P* = 0.03) has statistical differences. Although MR–Egger (OR, 1.19; 95% CI, 0.87–1.64) and weighted mode (OR, 1.28; 95% CI, 0.84–1.94) are similar to the estimated values of IVW in magnitude, they do not achieve statistical significance. This may be attributed to the limited efficacy, warranting further more favorable analyses to establish the potential causal relationship.

## 4 Discussion

To our knowledge, our study represents the first MR analyses aimed at investigating the causal relationship between body water content and kidney function at the genomic level. Previous studies on the relationship between body water content and kidney function in CKD patients mainly relied on BIA technology, offering insights into fluid status. These cross-sectional studies could not explain the causal relationship between excessive body water content and kidney function decline (3, 4). However, our study is based on the novel and large amount of GWAS summary data to predict the casual relationship between body water content and the development of CKD. Our results obtained by MR analyses are resilient against causal inversion and avoid the effect of confounding factors to a great extent. Moreover, this study lies in the utilization of the two-sample MR analyses method, involving a large number of summarized genetic data from UK Biobank and CKDGen consortium, which effectively avoided the overlap of samples. Therefore, our results are more reliable and may provide some guidance for the prevention, treatment, and management of CKD in the future.

The edematous status significantly influences the kidney outcomes in CKD patients. Previous studies have employed various markers of fluid status, with many relying on the ECW/TBW and the level of over-hydration measured by BIA (29, 30). The mechanisms underlying CKD progression caused by excessive body water content have been described, including factors such as the decrease of renal blood flow caused by the increase of renal efferent pressure, glomerular sclerosis, endothelial cell activation, and renal inflammation (31, 32). Compared with patients or animals without excessive body water content, those with excessive body water content exhibit more macrophage infiltration and increased expression of pro-inflammatory cytokines (such as tumor necrosis factor-α and interleukin-6) (31). Additional studies have shown that excessive body water content is related to the severity of anemia and an augmented risk of cardiovascular morbidity in CKD patients (3, 33).

Some observational studies have shown that excessive body water content will lead to the decline of kidney function among CKD patients. Low et al. demonstrated that excessive body water content in CKD patients with type 2 diabetes mellitus was independently related to the progression of CKD, suggesting that body water content played a pivotal role in the deterioration of kidney function (4). Moreover, excessive body water content has been identified as an independent risk factor for both renal replacement therapy and rapid decline of eGFR in patients with advanced CKD (14).

In our MR analysis, most of the participants in UK Biobank and CKDGen are Europeans, which effectively reduces the bias of population stratification. We systematically analyzed the causal relationship between genetically predicted BWM and kidney function. Our results reveal that the increase of BWM is correlated with the decrease of eGFR and UACR. Furthermore, an increase in BWM emerges as a risk factor for CKD stages 3–5 and rapid progress to CKD (CKDi25). Given the observed heterogeneity across the data, we advocate for the adoption of the random-effect model in the IVW analysis to enhance the robustness of our results. In the analysis of pleiotropy, directional pleiotropy was discerned solely in the data concerning BWM and UACR. Utilizing MR-PRESSO to detect and remove 13 outlier SNPs, the subsequent MR analyses revealed a robust causal effect between BWM and UACR. In various sensitivity analyses, the impact of BWM on kidney function exhibits consistent magnitudes. Therefore, our MR analyses affirm that excessive body water content is intricately linked to impaired kidney function.

In this study, we identified that the BWM gene SNP rs2005172 appears to have an impact on the pathological progression of renal fibrosis. The specific mechanism involves impairing glomerular permeability, stimulating epithelial–mesenchymal transition of podocytes, and promoting the expression of the fibrosis-associated classic molecule - transforming growth factor β (34, 35). Meanwhile, rs35874463 has also been confirmed to enhance inflammatory responses and increase the transcription levels of the fibrosis signaling pathway transforming growth factor β/smads, thereby exacerbating renal fibrosis (36, 37). Furthermore, we have found that rs165656 may exert its influence on kidney function by modulating dopamine metabolism, mediating Na^+^, K^+^-ATPase activity, disrupting the processes of diuresis and natriuresis, and consequently impacting overall renal function (38, 39). The findings of these studies confirm that SNPs associated with BWM may potentially influence the renal function of patients with kidney diseases through other pathophysiological mechanisms.

BIA, as a non-invasive, label-free, and quantitative detection technology, has great advantages for physiological and pathological analysis of tissues. It proves beneficial for the early identification of changes in fluid, allowing for a systematic clinical assessment of edema status and facilitating the management of target weight. An increasing number of studies have recognized the monitored whole-body water mass through BIA technology as a marker for evaluating the volume status of patients with CKD. However, whether BIA technology provides an appropriate volume status marker remains controversial. This value may be affected by age, sex, nutritional status, obesity, inflammation and muscle mass. Especially for patients with nephropathy, in patients with obvious fluid overload and muscle mass loss, bioelectrical impedance technique cannot distinguish extravascular ECW from plasma ECW. Future investigations should aim to identify a more accurate tool for evaluating the volume status or combining other biological indicators, so as to carry out more reliable analyses.

Several limitations should be acknowledged. First, this study mainly focuses on the European population, and the causal effects in other demographic groups need to be re-verified. Secondly, due to summary-level data, non-linear relationships or stratification effects are not captured. Finally, the evaluation of edematous status is very important for elucidating the causal relationship with exposed genes.

## 5 Conclusion

Genetic predictions indicate a causal effect between excessive body water content and increased eGFR and albuminuria levels, as well as an increased risk of CKD. This MR study undertook a thorough and detailed analysis of body water content and kidney function, providing novel insights for the prevention, treatment, and management of CKD.

## Data availability statement

Data for this study come from the UK Biobank and the CKDGen consortium. No original data were collected for the MR study.

## Ethics statement

Ethical approval was not required for the study involving humans in accordance with the local legislation and institutional requirements. Written informed consent to participate in this study was not required from the participants or the participants’ legal guardians/next of kin in accordance with the national legislation and the institutional requirements. Ethical approval for each of the studies included in the investigation can be found in the original publications.

## Author contributions

XW: Conceptualization, Data curation, Validation, Writing – original draft. ML: Data curation, Methodology, Validation, Writing – review & editing. ZF: Data curation, Methodology, Validation, Writing – review & editing. YH: Funding acquisition, Project administration, Writing – review & editing. LY: Project administration, Visualization, Writing – review & editing. ZQ: Writing – review & editing. YD: Conceptualization, Funding acquisition, Investigation, Writing – review & editing.
