# Supplementary material for: Whole-body water mass and kidney function: a Mendelian randomization study
Source: Front Endocrinol (Lausanne). 2024 Apr 3;15:1336142. doi: 10.3389/fendo.2024.1336142 (PMC11022284; doi:10.3389/fendo.2024.1336142)

Supplementary Material

**Supplementary Figure 1.** Leave-one-out plots of significant and nominal significant estimates from genetically predicted BWM on (a) eGFR; (b) UACR; (c) CKD stage 3-5; (d) CKDi25.

**a.**





**b.**


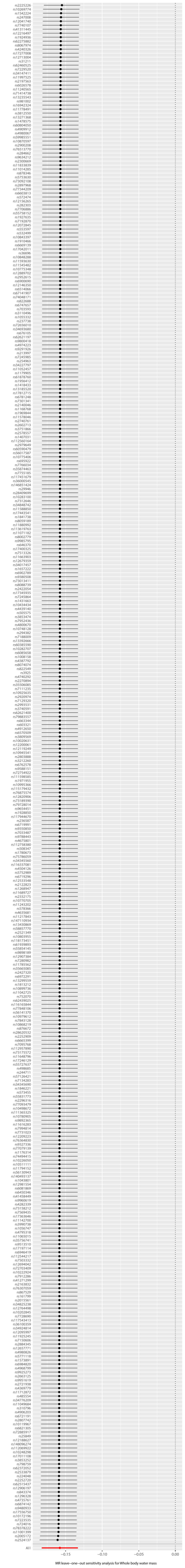


**c.**


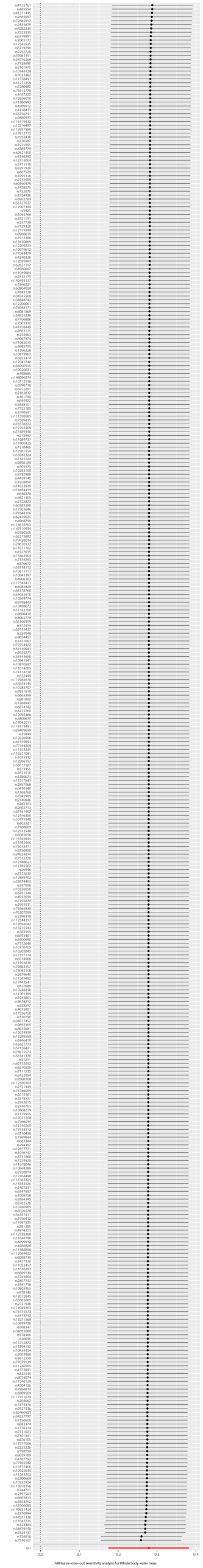


**d.**


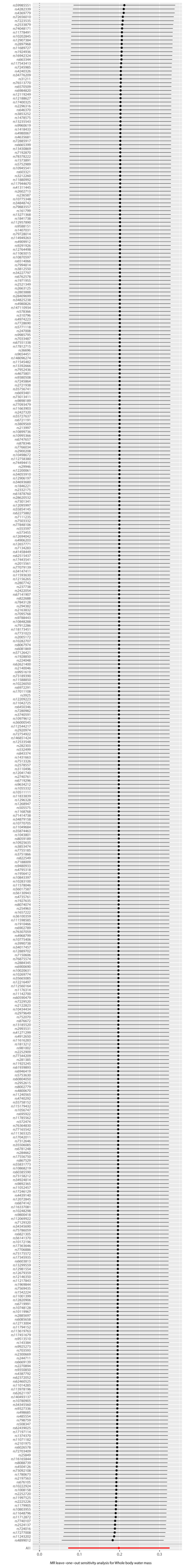


**Supplementary Figure 2. Funnel plots of significant and nominal significant estimates from genetically predicted BWM on (a) eGFR; (b) UACR; (c) CKD stage 3-5; (d) CKDi25.**


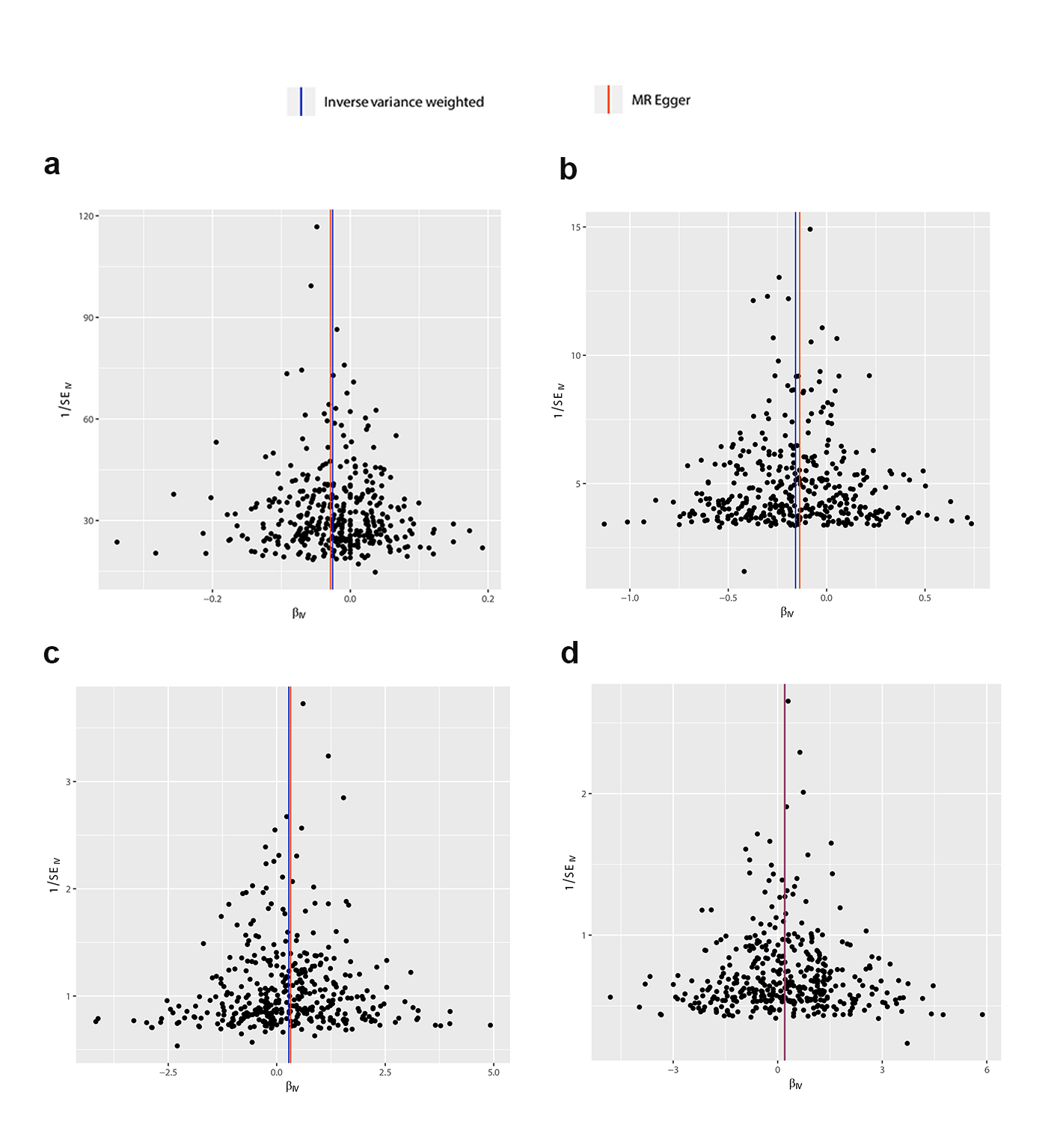

Supplement: Supplementary file 2 [file DataSheet_1.docx]
